# Evaluation of the relationship between effervescent paracetamol and blood pressure: clinical trial

**DOI:** 10.1186/s12872-015-0161-7

**Published:** 2015-12-10

**Authors:** Mencia Benitez-Camps, Ernest Vinyoles-Bargalló, Oriol Rebagliato-Nadal, Rosa Morros-Pedrós, Helena Pera-Pujadas, Antoni Dalfó-Baqué, Ignacio López-Pavón, Carlos Roca-Sánchez, Rosa Maria Coma-Carbó, Mariano De La Figuera Von Wichmann, Lucas Mengual-Martínez, Carmen Yuste-Marco, Montserrat Teixidó-Colet, Josep M. Pepió i Vilaubí, Riera Ciurana-Tost, Rosa Pou-Vila, Ma Antònia Vila-Coll, Josep Maria Bordas-Julve, Rosa Aragonès-Forès, Francisco Javier Pelegrina-Rodríguez, Josep Agudo-Ugena, Carlos Blanco-Mata, Jon de la Iglesia Berrojalbiz, Natalia Burgos-Alonso, Maria Cruz Gómez-Fernández

**Affiliations:** CAP Gòtic, Institut Català de la Salut, Barcelona, Spain; CAP La Mina, Institut Català de la Salut, Barcelona, Spain; Institut Universitari d’Investigació en Atenció Primària Jordi Gol, Barcelona, Spain; Departament de farmacologia i terapèutica Universitat Autònoma de Barcelona, Barcelona, Spain; CAP Santa Coloma de Gramenet, Institut Català de la Salut, Barcelona, Spain; CAP El Maresme, Institut Català de la Salut, Barcelona, Spain; CAP Sardenya, Barcelona, Spain; CAP Badia, Institut Català de la Salut, Badia del Vallès, Barcelona Spain; CAP Baix Ebre, Institut Català de la Salut, Tarragona, Spain; CS Mina del Morro, País Vasco, Spain; CS Bolueta, País Vasco, Spain; Clinical Trial Monitoring, Pais Vasco, Spain; Clinical Trial Monitoring and Coordination, País Vasco, Spain

**Keywords:** Hypertension, Effervescent paracetamol, Control

## Abstract

**Background:**

Paracetamol’s solubility is achieved by adding to the excipient sodium salts, either as bicarbonate, carbonate or citrate. As the relationship between salt and hypertension is well known, due to the sodium content it has raised a hypothesis that may interfere with the control of that risk factor.

Therefore, the objective of this study is to evaluate the effect on blood pressure of effervescent paracetamol compared to non-effervescent, in hypertensive patients.

**Methods/Design:**

This is the protocol of a phase IV multicenter clinical trial, randomized, controlled, crossover, open, which will compare the effect of two different formulations of paracetamol (effervescent or non-effervescent) in the blood pressure of hypertensive patients, with a seven weeks follow up.

49 controlled hypertensive patients will be included (clinical BP lower than 150 and 95 mmHg, and lower than 135 mmHg and 85 mmHg in patients with diabetes or a history of cardiovascular event, and daytime ambulatory measurements lower than 140 and 90 mmHg) and mild to moderate pain (Visual Analog Scale between 1 and 4).

The study was approved by the ethics committee of the Fundació Jordi Gol i Gurina and following standards of good clinical practice.

The primary endpoint will be the variations in systolic BP in 24 h Ambulatory Blood Pressure Monitoring, considering significant differences 2 or more mmHg among those treated with non-effervescent and effervescent formulations.

Intention-to-treat and per-protocol analysis will be held.

**Discussion:**

Despite the broad recommendation not to use effervescent drugs in patients with hypertension, there are relatively little studies that show exactly this pressor effect due to sodium in salt that gives the effervescence of the product.

This is the first clinical trial designed to study the effect of effervescence compared to the non-effervescent, in well-controlled hypertensive patients with mild to moderate pain, performed in routine clinical practice

**Trial registration:**

NCT 02514538

## Background

Hypertension control has become a prime target because of its significant relationship to cardiovascular disease (1–3). At the same time, musculoskeletal diseases are among the leading causes of analgesics prescription (4).

It is known that the continued use of nonsteroidal anti-inflammatory drugs (NSAIDs) carries a high risk of side effects, including difficulty in controlling hypertension (5–7). Therefore paracetamol seems the best option for hypertensive patients requiring analgesia (7).

However, in some studies it has been observed that paracetamol can also be associated with an increase in BP, increasing the risk of hypertension or hindering its control, although there is no consensus on this issue (8–13).

Moreover, one of the most common forms of presentation of paracetamol is soluble, which appears to increase the absorption rate, reduce the time of onset of action and facilitates its administration when there are intake difficulties.

Solubility of paracetamol is achieved by adding to the excipient salts containing sodium, either as bicarbonate, carbonate or citrate. As the relationship between salt and hypertension is well known, due to the sodium content it has raised a hypothesis about whether these presentations could interfere in the control of hypertensive patients or favor the emergence of new cases of hypertension. There are some observational studies, although few patients included, which seem to support this relationship between consumption of effervescent formulations and the difficulty of hypertension control (14, 15)

Such small samples, the potential confounders not included in the studies such as patients’ pain, the possible phenomenon of regression to the mean, and data from other studies that contradict this hypothesis (16–21) make the existence of more studies needed, with the express purpose of seeing the effect of effervescent paracetamol on BP.

## Methods/Design

### Objectives and hypothesis

#### Objectives

The overall aim of the study is to evaluate the pressor effect of effervescent paracetamol formulation compared to the formulation of non-effervescent paracetamol in hypertensive patients.

#### Hypothesis

The effervescent preparations normally don’t contain sodium chloride (salt directly related to BP), but contain other salts. So, we hypothesize that hypertensive patients treated with effervescent paracetamol should not present BP higher than those taking formulations non-effervescent.

### Study design

This is a multicenter, randomized, controlled, crossover, open, phase IV clinical trial, which compares the effect of two different formulations of paracetamol (effervescent or non-effervescent tablets) in the blood pressure of hypertensive patients after 3 weeks treatment (coded EUDRACT 2010-023485-53). The washout period between the two treatment periods is approximately 1 week (minimum 3 days).

### Setting

The study will be conducted in Primary Care, with the participation of 13 primary care centers in Catalonia and Euskadi. The study protocol was approved by the Ethics Committee of IDIAP Jordi Gol and the rules of good clinical research practice will be followed.

### Study population

#### Inclusion criteria

Patients included in the study must meet the following criteria:

- be hypertensive patients over 18 years
- with chronic osteoarticular disease
- which regularly need analgesic treatment.

They must submit BP in the consultation lower than 150 and 95 mmHg or lower than 135 and 85 mmHg if they have associated cardiovascular disease (stroke, coronary heart disease, peripheral arterial disease) or diabetes mellitus.

Antihypertensive treatment must be stable and unchanged in the last month, or patients should be controlled without drugs. In relation to chronic osteoarticular disease, they should have a degree of mild to moderate pain, with a score between 1 and 4 on a VAS.

#### Exclusion criteria

Patients with allergy, intolerance or contraindication to paracetamol or tramadol will be excluded, as those who have taken NSAIDs orally or parenterally in the last week, or have a high degree of pain (VAS > 4) or poorly controlled hypertension (office BP > 150/95 mmHg or daytime ambulatory mean BP > 140/90 mmHg).

Patients with heart failure due to systolic and / or diastolic dysfunction will also be excluded, those who have suffered a cardiovascular event (myocardial infarction, unstable angina or stroke of any type) in the last 6 months, presenting sleep apnea or any form of secondary hypertension, elevated transaminases (higher than 3 times normal value), or a glomerular filtration rate <30 ml/min, over a maximum period of three months before starting the study; patients with dementia or judicial disability, with alcoholism or other addictions; pregnant patients; patients treated with oral anticoagulants or subcutaneous heparin. Patients in which changes are foreseen in usual dose drugs with effects on BP throughout the study (alpha blockers, tricyclic antidepressants, beta blockers in eye drops, sympathomimetic vasoconstrictor, other effervescent agents, hormonal contraceptives, NSAIDs, corticosteroids, anabolic, erythropoietin, cyclosporine) or those who will initiate major changes in lifestyles (onset or increase physical exercise, dietary changes); those who do not give their informed consent and that in the opinion of the investigator, have poor adherence or may become lost to follow-up.

### Recruitment process

Patients meeting the inclusion criteria will be identified using the electronic medical record systems, OSABIDE in Osakidetza and the SISAP in the Catalan Health Service. Considering the information in the medical record, each doctor will check the list of candidate patients, rejecting those that a priori meet any of the exclusion criteria, that is, cases in which inappropriate patients have been identified by the electronic selection’ process.

Participating physicians may also include patients who met inclusion criteria, but for some reason were not selected by the selection system.

Once elected, it will be proposed to patients to participate in the trial, giving writing information and requesting written informed consent, after a first visit for confirmation of all the inclusion criteria and none of exclusion.

In addition, a blood test assessing complete blood count, kidney and liver function will be performed to all patients who do not have one including all these parameters in the previous 3 months.

### Washout period

A 3 to 7 days washout period for paracetamol will precede randomization. During this washout period, the analgesic treatment will be tramadol when necessary.

### Randomization

Patients who met all inclusion criteria and none of the exclusion will be assigned through centralized, automatic and masked randomization, for successive scrambling. Patients will be allocated through an electronic case report form (CRF) to the first of two treatment sequences to follow: AB or BA.

- A: Effervescent Paracetamol (Termalgin ® envelopes) at doses of 1000 mg / 8 h
- B: Paracetamol in non-effervescent tablets (Kern pharma ® Paracetamol 1 g), at doses of 1000 mg / 8 h.

### Intervention

The initial paracetamol dose is 1000 mg every 8 h. If the pain is not controlled (VAS > 3), tramadol will be administered at doses between 50 i 150 mg / day, at the discretion of the investigator.

Rescue analgesia chosen is tramadol, following the NICE Osteoarthritis Guide recommendations, which ranks in the second therapeutic step (4).

The medications in this trial are marketed and will be procured in Spain and conditioned (labeled only for use in clinical trials) according to the standards of Good Manufacturing Practice for medicinal products.

To assess compliance, counting tablets or sachets dispensed will be held in each subsequent visit to dispensing medication. It will be considered adequate if it is between 80 and 110 %.

BP must be measured in both arms at first visit, subsequent blood pressures measures will be performed in the arm with the higher reading. These measures must be done with the person seated and standing, and using an appropriate cuff size for the person’s arm. For ambulatory blood pressure monitoring (ABPM) validated SpaceLabs 90207 (22) or Microlife Watch BP (23) devices will be used, and will be considered valid those with 60 % or more valid readings, or at least 20 daytime readings and 8 nocturnal, as recommended by the latest guidelines (24).

**Fig. 1:**
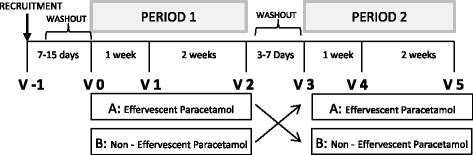
Flow chart

**Fig. 2:**
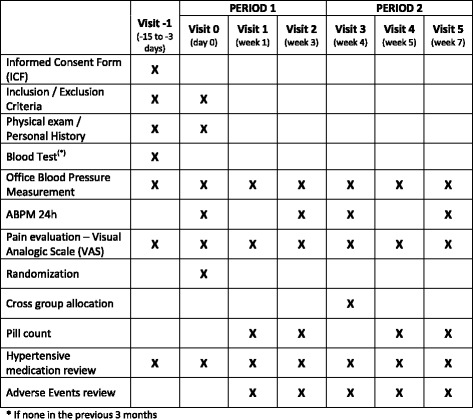
Visit schedule. * If none in the previous 3 months

Figures 1 and 2 show the flow chart diagram and visit schedule.

### Evaluation of results

#### Primary endpoint

The primary endpoint is the change in mean daytime, night-time (sleep) and 24-h systolic BP, measured by ABPM, from baseline at 3 weeks of treatment in both periods.

#### Secondary endpoints

- Changes in systolic BP measured in the clinic at the end of 3-week follow-up respect baseline in both periods.
- 24-h, daytime and night-time (sleep) diastolic BP measured by ABPM: change from baseline at 3 weeks of treatment in both periods
- Changes in diastolic BP measured in the clinic at the end of 3-week follow-up respect baseline in both periods.
- Percentage of patients maintaining clinical BP under 140 and 90 mmHg at the end of each period
- Degree of pain assessed by visual analog scale
- Consumption of rescue medication
- Therapeutic compliance

#### Adverse effects

Mild adverse events occurring during the study will be collected in the case report form, indicating the likelihood that may be related to drugs; if serious adverse reactions occur they will be immediately notified to the responsible for pharmacovigilance.

### Sample size

49 patients are required to detect a difference in systolic BP equal to or greater than 2 mmHg in 24-h systolic BP detected by ABPM (minimum difference with relevance in clinical practice), with a standard deviation of the mean difference of 4.5 mmHg (14), and for a crossover design. An alpha error of 5 % and beta error of 20 % in a two-tailed test, and maximum loss rate of 15 % is assumed.

### Statistical analyses

The analysis will be carried out on an intention-to-treat basis, comparing the mean blood pressure at the end of the period with that observed at initial time, adjusting for the baseline blood pressure at the start of each period in all patients which at least have received one dose of treatment. Moreover, we will perform a per protocol analysis with only the patients who have not changed their treatment within the 7-weeks of the trial and have not been withdrawn.

Categorical variables will be presented using frequencies and percentages whereas that continuous variables will be presented using the mean, median, standard deviation, minimum, maximum, interquartile range and number of subjects under observation. All statistical tests are going to be two-tailed tests with an α level of 0.05, obtaining confidence intervals of 95 %.

To analyze the differences between drugs in BP change between the beginning and the end of treatment, an ANOVA model will be used with the period, sequence, treatment and subject factors, declaring random factor subject nested within sequence. From this model estimators of the difference between the two formulations with confidence intervals to 95 % will be obtained.

For all other variables the following strategy is to be used: to compare categorical variables intragroup McNemar’s test will be used, for continuous variables dependent Student’s *t*-test. If not met the assumptions of applicability, nonparametric methods (Wilcoxon test) will be used.

We will adjust for potential confounding or modifying variables: age, sex, comorbidity as diagnosis of dyslipidemia, diabetes, stroke, Peripheral artery disease or myocardial infarction.

In the mixed-effect analysis, all the data available from each of the subjects included in the study will be considered, regardless of whether there is missing data. Values of missing data will not be imputed, except in the case of the baseline values for the second period, in which case they will be assigned the same value as the baseline measurements at the start of the study. It has been shown that this is more robust than other approaches to dealing with missing data.

#### Data quality and management

The study will be subject to regular monitoring by the Clinical Research Associate (CRA), personnel specially trained in Good Clinical Practice standards. The CRA will monitor the patient recruitment and ethical issues; furthermore, he will guarantee the quality of data and ensure the patient safety. In addition to this, the CRA will revise the incidence of adverse events and concomitant medication. Data will be evaluated following the protocol and in concordance with source documents.

#### Legal and ethical considerations

This clinical trial will comply with the following regulations: the 2008 version of the Declaration of Helsinki, the Spanish Royal Decree 223/2004 of 6th February and the recommendations of the Council of Europe (Good Clinical Practices for Clinical Trials and Medicinal Products in the European Community, 17th January 1997). The protocol of the clinical trial has been agreed on by the primary care research committee, approved by the Clinical Research Ethics Committee of Jordi Gol i Gurina, which is the reference committee, and authorized by the Spanish Agency for Medicaments and Health Products.

Only the researchers involved in the study will have access to patient codes. In relation to this, we will comply with the Spanish Act 14/2007 of Biomedical Research and Royal Decree 1720/2007 of 21st December that approves the regulations on the Development of the Organic Act 15/1999 of 13th December on Personal Data Protection.

### Limitations

The main limitation of the study is that it is an open trial, since it is not possible to have an effervescent placebo, but this has been attempted to solve by the blinding of data analysis.

Another limitation is the inability to adjust the results for dietary salt intake, or exercise, a fact that has been attempted to solve by explaining and emphasizing the need not to change lifestyle habits during the study period.

## Discussion

Among the non-pharmacological measures for hypertension treatment, in all existing clinical practice guidelines it is recommended to reduce salt intake (25–28), referring to the decline in consumption sodium chloride. However, there is controversy over whether this effect on BP may be produced for any salt containing sodium. From here, sometimes the recommendation made is not to reduce salt intake, but to reduce sodium intake, and this has led to recommend to hypertensive patients to avoid consuming effervescent drugs. But, despite the broad recommendation not to use effervescent drugs in patients with hypertension, there are hardly any studies that show exactly this pressor effect attributable to sodium in salt that gives the effervescence of the product. So far existing trials are quasi-experimental studies with a Before and After design, where the drugs involved were not only paracetamol but also ibuprofen or acetylcysteine (14), having some of these drugs already in itself a pressor effect, or are observational studies (15), which do not allow establishing a cause and effect relationship, or are made in laboratory conditions, or are poorly extrapolated to the conditions of everyday life (16–20). In addition, in some of them it is not clear whether they have taken into account some confounding variables, such as the decrease in pain after the start of analgesic treatment (14).

Although there is a case–control study that evaluates the effect of effervescent drugs in the risk of developing cardiovascular events, which results are in favor of increased risk (29), this would be the first clinical trial with the primary objective of evaluating the effect of effervescent paracetamol in hypertensive patients.

As it is executed in routine clinical practice, this will help to extrapolate the results to include the general population.

The only drugs not allowed are those who already have a recognized pressor effect as NSAIDs or oral corticosteroids. Besides, there is no increase in BP due to changes in antihypertensive medication because this is an exclusion criterion.

The results will have high validity due to performing BP measurements not only in consultation, but also through ABPM, thereby minimizing the possible white coat effect which could appear with measurements taken by medical personnel. This is also a relevant difference with studies already existing.

## Trial status

At this time, patient recruitment is proceeding to complete.

## Abbreviations

BP: Blood Pressure
AVS: Analogic visual scale
ABPM: lAmbulatory blood pressure monitoring
HT: Hypertension
NSAID: Non-steroidal anti-inflammatory drugs
CRF: Case report form
CRA: Clinical research associate

## References

[1] Kearney PM, Whelton M, Reynolds K, Muntner P, Whelton PK, He J (2005). Global burden of hypertension: analysis of worldwide data. Lancet.

[2] Banegas JR (2005). Epidemiología de la hipertensión arterial en España. Situación actual y perspectivas. Hipertensión.

[3] Ezzati M, Hoorn SV, Rodgers A, Lopez AD, Mathers CD, Murray CJ (2003). Estimates of global and regional potential health gains from reducing multiple major risck factors. Lancet.

[4] National Institute for health and clinical Excellence (2008). Osteoarthritis: national clinical guideline for care and management in adults.

[5] Frishman WH (2002). Effects of nonsteroidal anti-inflammatory drug therapy on blood pressure and peripheral edema. Am J Cardiol.

[6] Chalmers JP, West MJ, Wing LM, Bune AJ, Graham JR (1984). Effects on indomethacin, sulindac, naproxen, aspirin and paracetamol in treated hypertensive patients. Clin Exp Hypertens A.

[7] Radack KL, Deck CC, Bloomfield SS (1987). Ibuprofen interferes with the efficacy of antihypertensive drugs. A randomized, double-blind, placebo-controlled trial of ibuprofen compared with acetaminophen. Ann Intern Med.

[8] Forman JP, Rimm EB, Curhan GC (2007). Frequency of analgesic use and risk of hypertension among men. Arch Intern Med.

[9] Kurth T, Hennekens CH, Stümer T, Sesso HD, Glynn RJ, Buring JE (2005). Analgesic use and risk of subsequent hypertension in apparently healthy men. Arch Intern Med.

[10] Forman JP, Stampfer MJ, Curhan GC (2005). Non-narcotic analgesic dose and risk of incident hypertension in women. Hypertension.

[11] Dedier J, Stampfer MJ, Hankinson SE, Willett WC, Speizer FE, Curhan GC (2002). Nonnarcotic analgesic use and the risk of hypertension in US women. Hypertension.

[12] Turtle EJ, Dear JW, Webb DJ (2013). A systematic review of the effect of paracetamol on blood pressure in hypertensive and non-hypertensives subjects. BJCP.

[13] Dawson J, Fulton R, McInnes GT, Morton R, Morrison D, Padmanabhan S (2013). Acetaminophen use and change in blood pressure in a hypertensive population. J Hypertens.

[14] Ubeda A, Llopico J, Sánchez MT (2009). Blood pressure reduction in hypertensive patients after withdrawal of effervescent medication. Pharmacoepidemiol Drug Saf.

[15] Bendahan Barchilon G, Lizano Díez I, Rodríguez Pérez E, Cortes Pérez PJ, Sorribes Capdevila M, Modamio CP (2007). El sodio como excipiente de los medicamentos podría relacionarse con elevación de la presión arterial en pacientes hipertensos. FAP.

[16] Sharma AM, Kribben A, Schattenfroh S, Cetto C, Distler A (1990). Salt sensivity in humans is associated with abnormal acid base regulation. Hypertension.

[17] Luft FC, Steinberg H, Ganten U, Meyer D, Gless KH, Lang RE (1988). Effect of sodium chloride and sodium bicarbonate on blood pressure in stroke-prone spontaneously hypertensive rats. Clin Sci (Lond).

[18] Shore AC, Markandu ND, MacGregor GA (1988). A randomized crossover study to compare the blood pressure response to sodium loading with and without chloride in patients with essential hypertension. J Hypertens.

[19] Luft FC, Zemel MB, Sowers JA, Fineberg NS, Weinberger MH (1990). Sodium bicarbonate and sodium chloride: effects on blood pressure and electrolyte homeostasis in normal and hypertensive man. J Hypertens.

[20] Schorr U, Distler A, Sharma AM (1996). Effect of sodium chloride- and sodium bicarbonate-rich mineral water on blood pressure and metabolic parameters in elderly normotensive individuals: a randomized double-blind crossover trial. J Hypertens.

[21] Sejoong K, Yang JY, Jung ES, Lee J, Heo NJ, Lee JW (2014). Effects of sodium citrate on salt sensitivity and kidney Injury in chronic renal failure. JKMS.

[22] O’Brien E, Mee F, Atkins N, O’Malley K (1991). Accuracy of the SpaceLabs 90207 determined by the British Hypertension Society Protocol. J Hypertens.

[23] Ragazzo F, Saladini F, Palatini P (2010). Validation of the Microlife WatchBP 03 device for clinic, home and ambulatory blood pressure measurement, according to the International Protocol. Blood Pressure Monitoring.

[24] Parati G, Stergiou G, O’Brien E, Asmar R, Beilin L, Bilo G (2014). European Society of Hypertension practice guidelines for ambulatory blood pressure monitoring. J Hypertens.

[25] Chobanian AV, Bakris GL, Black HR, Cushman WC, Green LA, Izzo JL (2003). The seventh report of the Joint National Committee on Prevention, Detection, Evaluation, and Treatment of High Blood Pressure: the JNC 7 Report. JAMA.

[26] Mancia G, Fagard R, Narkiewicz K, Redon J, Zanchetti A, Böhm M (2013). 2013 ESH/ESC guidelines for the management of arterial hypertension: the Task Force for the Management of Arterial Hypertension of the European Society of Hypertension (ESH) and of the European Society of Cardiology (ESC). Eur Heart J.

[27] Dasgupta K, Quinn RR, Zarnke KB, Rabi DM, Ravani P, Daskalopoulou SS (2014). The 2014 Canadian hypertension education program recommendations for blood pressure measurement, diagnosis, assessment of risk, prevention, and treatment of hypertension. Can J Cardiol.

[28] The clinical management of primary hypertension in adults CG127. http://www.nice.org.uk/guidance/cg127.

[29] George J, Majeed W, Mackenzie IS, Macdonald TM, Wei L (2013). Association between cardiovascular events and sodium-containing effervescent, dispersible, and soluble drugs: nested case–control study. BMJ.

